# Data Mining and Biochemical Profiling Reveal Novel Biomarker Candidates in Alzheimer’s Disease

**DOI:** 10.3390/ijms26157536

**Published:** 2025-08-04

**Authors:** Annamaria Vernone, Ilaria Stura, Caterina Guiot, Federico D’Agata, Francesca Silvagno

**Affiliations:** 1 Department of Neurosciences, University of Turin, Via Cherasco 15, 10125 Torino, Italy; annamaria.vernone@unito.it (A.V.); ilaria.stura@unito.it (I.S.); caterina.guiot@unito.it (C.G.); 2 Department of Oncology, University of Turin, Via Santena 5 bis, 10126 Torino, Italy

**Keywords:** Alzheimer’s Disease, iron, mitochondrial metabolism, biomarkers, data mining, amino acid composition

## Abstract

The search for the biomarkers of Alzheimer’s disease (AD) may prove essential in the diagnosis and prognosis of the pathology, and the differential expression of key proteins may assist in identifying new therapeutic targets. In this proof-of-concept (POC) study, a new approach of data mining and matching combined with the biochemical analysis of proteins was applied to AD investigation. Three influential online open databases (UniProt, AlzGene, and Allen Human Brain Atlas) were explored to identify the genes and encoded proteins involved in AD linked to mitochondrial and iron dysmetabolism. The databases were searched using specific keywords to collect information about protein composition, and function, and meta-analysis data about their correlation with AD. The extracted datasets were matched to yield a list of relevant proteins in AD. The biochemical analysis of their amino acid content suggested a defective synthesis of these proteins in poorly oxygenated brain tissue, supporting their relevance in AD progression. The result of our POC study revealed several potential new markers of AD that deserve further molecular and clinical investigation. This novel database search approach can be a valuable strategy for biomarker search that can be exploited in many diseases.

## 1. Introduction

In the last thirty years, the worldwide prevalence of Alzheimer’s disease (AD) and other dementia among aged adults has increased by 1.6 times, associated with a longer lifespan [1]. This escalating trend strongly challenges the global healthcare system in terms of both mortality rates and disability care and urgently demands novel therapeutic and preventive approaches. In neurodegenerative diseases, particularly in AD, protein amyloid aggregation into insoluble fibrils is influenced by several environmental factors, such as ionic strength [2] and the accumulation of metals like calcium [3] and iron [4]. Although several oligo-elements are normally enzymatic cofactors essential for tissue physiology, their deregulation can cause toxicity and cell death, which strongly impacts brain architecture and function.

Iron is very abundant, and its metabolism is extremely critical, as it is a well-known oxidizing agent. It is normally deposited in cytoplasmic and mitochondrial ferritin, and mitochondrial frataxin, precisely to prevent its reactivity from leading to the production of free radicals and, in the brain, consequent oxidative stress and neuronal death [5,6,7]. Iron is widely used in the mitochondrial compartment, especially as a cofactor of the proteins of the respiratory chain; and therefore, its use must be carefully calibrated. As a cofactor, iron is bound to geometric organic structures both in iron–sulfur clusters and in heme. The latter form is the most abundant, as heme represents the prosthetic group of huge amounts of mitochondrial cytochromes; therefore, the metabolism of this molecule is potentially involved in iron deregulation; for example, the increase in free iron could stem from a deranged heme biosynthetic pathway or catabolism. The abnormal deposition of iron has been described as one of the leading causes of neurodegenerative diseases such as AD [8,9,10,11]. The neuroprotective properties of heme are essential for maintaining neuronal health and function [12], although heme accumulation can be one significant source of ROS and oxidative stress [13].

Another functional abnormality reported in AD and related to iron balance is the defective activity of the respiratory chain associated with oxidative phosphorylation (OXPHOS). Mitochondria represent a cellular powerhouse necessary for the synthesis of ATP, which is abundantly used in neurons and is indispensable for maintaining the ionic gradients that allow for electrical conduction. A lack of oxygen or mitochondrial defects (of genetic or environmental origin, such as iron accumulation) or oxidative stress can cause mitochondrial damage. Iron accumulation and mitochondrial damage are strictly linked and can be the cause of each other; they both lead to neuronal death, visible in AD [11,14,15,16,17,18,19].

The proteins modulated in the form of AD associated with iron and mitochondrial dysmetabolism are most probably related to heme synthesis, iron metabolism, or hypoxia. Because the biosynthesis of proteins depends on environmental cues that determine amino acid availability, the proteins that accumulate in poorly oxygenated tissue are low in glutamate (E) and rich in glutamine (Q), due to the activation of the enzyme glutamine synthetase in hypoxia [20]. On the contrary, proteins requiring more glutamate than glutamine are poorly synthesized in hypoxic tissues, such as the brain regions that are prone to neurodegenerative diseases, including AD. The E/Q ratio in the amino acid composition reveals the dependency of protein synthesis on oxygenation, as was established in skin and liver models, where a well-defined oxygenation gradient allowed for the analysis of specific proteins [21]. Although a clear anatomical separation of hypoxic and normoxic areas is not evident in the brain, the amino acid analysis could be proposed to support the results of the search for AD markers. In fact, proteins with a high E/Q value could be downregulated, and a low E/Q ratio could be prominent in proteins differentially expressed in AD.

Based on these considerations, we investigated the link between mitochondrial derangement, iron homeostasis, and AD. Starting from the assumption that mitochondrial damage can cause iron accumulation, and vice versa, defective iron utilization can induce mitochondrial damage, and both contribute to the development of AD, we wanted to establish which proteins related to mitochondrial metabolism, and in particular to mitochondrial iron handling, may be important players in AD.

The aim of this work was to identify the proteins associated with iron and mitochondrial dysfunction in AD by extracting and matching information from several different databases, excluding gene expression analysis. With this novel approach, we wanted to define a protein set that can contain new markers of AD, and stimulate their investigation in basic research, diagnostic evaluation, and follow-up studies.

We used three eminent online open databases to search for genes and the codified proteins involved in AD, focusing on iron-related and mitochondrial proteins. From the databases, we collected information about protein composition and function, and the meta-analysis data about their correlation with AD. Further biochemical analysis based on the results of published studies was integrated with the database search and spotlighted potential biomarkers of AD linked to iron homeostasis.

## 2. Results

### 2.1. Dataset Collection

We used online open databases to search for genes and the codified proteins involved in AD, focusing on iron-related and mitochondrial proteins. We selected three databases offering different information on proteins and their genetic code. On one hand, to gather data on proteins related to AD, we investigated the UniProt database, which is a bioinformatics resource containing protein sequence information and functional annotations that align with the FAIR (Findable, Accessible, Interoperable, Reusable) principles, thus facilitating the sharing, integration, and reuse of protein features. On the other hand, we took into consideration the genetics of AD and we searched the AlzGene database, which is a publicly available repository that comprehensively catalogs all genetic association studies in the field of AD [22]. The third search was carried out in the Allen Human Brain Atlas (AHBA) database, a multimodal atlas of gene expression and anatomy comprising an ‘all genes–all structures’ array-based dataset of gene expression and complementary in situ hybridization (ISH) gene expression studies targeting selected genes in specific brain regions [23]. Finally, additional research in PubMed and biochemical analysis supported the selection of proteins promising as novel biomarkers of AD.

#### 2.1.1. UniProt Investigation Results

In the first step, we investigated UniProt Disease, a curated resource within UniProt that provides comprehensive information on proteins associated with human disease and their genetic variants. We downloaded three proteins associated with early onset AD (EOAD): amyloid-beta precursor protein (Gene APP), presenilin-1 (Gene PSEN1), and presenilin-2 (Gene PSEN2); and three proteins associated with late onset AD (LOAD): apolipoprotein E (Gene APOE), disintegrin and metalloproteinase domain-containing protein 10 (Gene ADAM10), and phospholipid-transporting ATPase ABCA7 (Gene ABCA7).

In the second step, we explored UniProt by keyword search. We started with the general keyword “Alzheimer” and downloaded a dataset of 236 proteins (henceforth named UniProt A); then, we focused our search on more stringent keywords, with “Alzheimer AND iron”, and we obtained 25 proteins (henceforth named UniProt AI), and with the most specific keywords “Alzheimer AND iron AND mitochondrial”, we downloaded 16 proteins (henceforth named UniProt AIM). In this last dataset, the analysis of protein function was carried out in publications retrieved in PubMed with the search “protein name AND Alzheimer AND (iron OR mitochondrial)”. The investigation highlighted the relevance of three proteins in AD:

- Phosphatidylinositol-binding clathrin assembly protein (PICALM, gene: PICALM) acts as a modulator of the internalization of the transferrin receptor, thus modulating the entry of iron into cells [24];
- Appoptosin, also called mitochondrial glycine transporter (gene: SLC25A38), is important for heme synthesis, as it transports the glycine necessary for the first reaction of heme biosynthesis into the mitochondria [25];
- Humanin (gene: MT-RNR2) is a very small peptide encoded by the mitochondrial genome. It is associated with a longer life, and has antioxidant and mitochondria protective functions [26]. Because its transcription and amino acid content differ from the rest of the nuclear encoded proteins, we did not include this peptide in further analysis.

We also analyzed Gene Ontology (GO) terms when UniProt was investigated with the keyword “Alzheimer”, to take into consideration the functional information on AD-related proteins provided by GO terms. The search with the single keywords “Alzheimer”, “iron” and “mitochondrial” in the GO terms retrieved 4 proteins by “Alzheimer” GO term, 8 proteins by “iron” GO term, and 34 proteins by “mitochondrial” GO term, which were listed in the protein dataset downloaded from UniProt, thus confirming that in our data mining strategy, we did not neglect any protein of interest.

As a wider approach, we enlarged our search and sought to reveal the proteins related not only to AD, but also other forms of neurodegeneration; therefore, we investigated UniProt with the keywords “Alzheimer AND Parkinson” and “Alzheimer AND Dementia”. We extracted a list of 20 proteins and a dataset of 31 proteins, respectively.

In the third step, we considered the relevance of heme metabolism in mitochondrial impairment related to AD. In fact, heme dysregulation is linked to neurological disorders and is mediated by many mechanisms [12]. For this reason, we analyzed the proteins involved both in heme synthesis and mitochondrial function, and the enzymes of the heme biosynthetic pathway. First, the search of UniProt with the keywords “heme synthesis AND mitochondrial” retrieved a list of 53 proteins. The biochemical analysis of their function described in UniProt integrated with data from publications found in PubMed with the search “protein name AND Alzheimer” allowed for the identification of three important proteins linked to the heme biosynthetic cycle and involved in AD:

- Mitoferrin-1 (gene: SLC25A37) is a mitochondrial iron transporter that specifically mediates iron uptake in developing erythroid cells, thereby playing an essential role in heme biosynthesis [27];
- Mitoferrin-2 (gene: SLC25A28) is a mitochondrial iron transporter ubiquitously expressed that mediates iron uptake in all tissues [28];
- Frataxin (gene FXN), is a high-affinity iron-binding partner for ferrochelatase that is capable of both delivering iron to ferrochelatase and mediating the terminal step in mitochondrial heme biosynthesis [29].
- The proteins most sensitive to oxygen, based on the E/Q ratio, were mitoferrin-2 (E/Q = 1062) and frataxin (E/Q = 1429). Because they require a well oxygenated tissue for their synthesis, these proteins are scarcely produced in the hypoxic brain and their downregulation can impair neuronal function, leading to neurodegenerative diseases. Next, to fully investigate the whole heme synthesis pathway, we searched the information available in UniProt about the eight single biosynthetic enzymes: ALA synthase, ALA dehydratase, porphobilinogen synthase, uroporphyrinogen-III synthase, uroporphyrinogen decarboxylase, coproporphyrinogen-III oxidase, protoporphyrinogen oxidase, and ferrochelatase (heme synthase). Their amino acid composition was downloaded from UniProt, and the E/Q ratio was calculated to discover which proteins were most sensitive to oxygen. The E/Q value was always greater than 1 for all enzymes, indicating that they all should be synthesized in an environment that needs to be well oxygenated. However, none of these enzymes were linked to AD; therefore, we excluded these proteins from our final analysis.

#### 2.1.2. AlzGene Investigation Results

The AlzGene database collects data from genetic association studies published in the field of AD, along with results of the meta-analyses.

The ten most promising AD biomarkers were downloaded from the database, and information about their function and composition was searched in UniProt with the search “protein name AND Alzheimer”.

#### 2.1.3. AHBA Investigation Results

The AHBA is an open-access online resource accessible via the Allen Brain Atlas data portal, containing brain microarray gene expression data obtained from post-mortem tissue samples of adult human donors [30]. The AHBA includes two complementary datasets: a multi-donor atlas of genome-wide gene expression profiles derived from anatomically precise brain samples, covering all major brain structures and accompanied by histology-based neuroanatomical annotations aligned to a 2D and 3D MRI space; and a series of in situ hybridization studies targeting the selected gene sets in specific brain regions of interest, conducted on both neurotypical and disease-affected brains. In our investigation, the discovery of proteins of interest described in the AHBA could lead to a future analysis of differential gene expression, and the spatial mapping of such genes could highlight brain regions particularly affected by iron and mitochondrial perturbation in AD. In this study, the database was used as a source of genes described as related to AD, and from this database, we identified and downloaded 28 proteins.

### 2.2. Dataset Matching and Analysis

**Figure 1:**
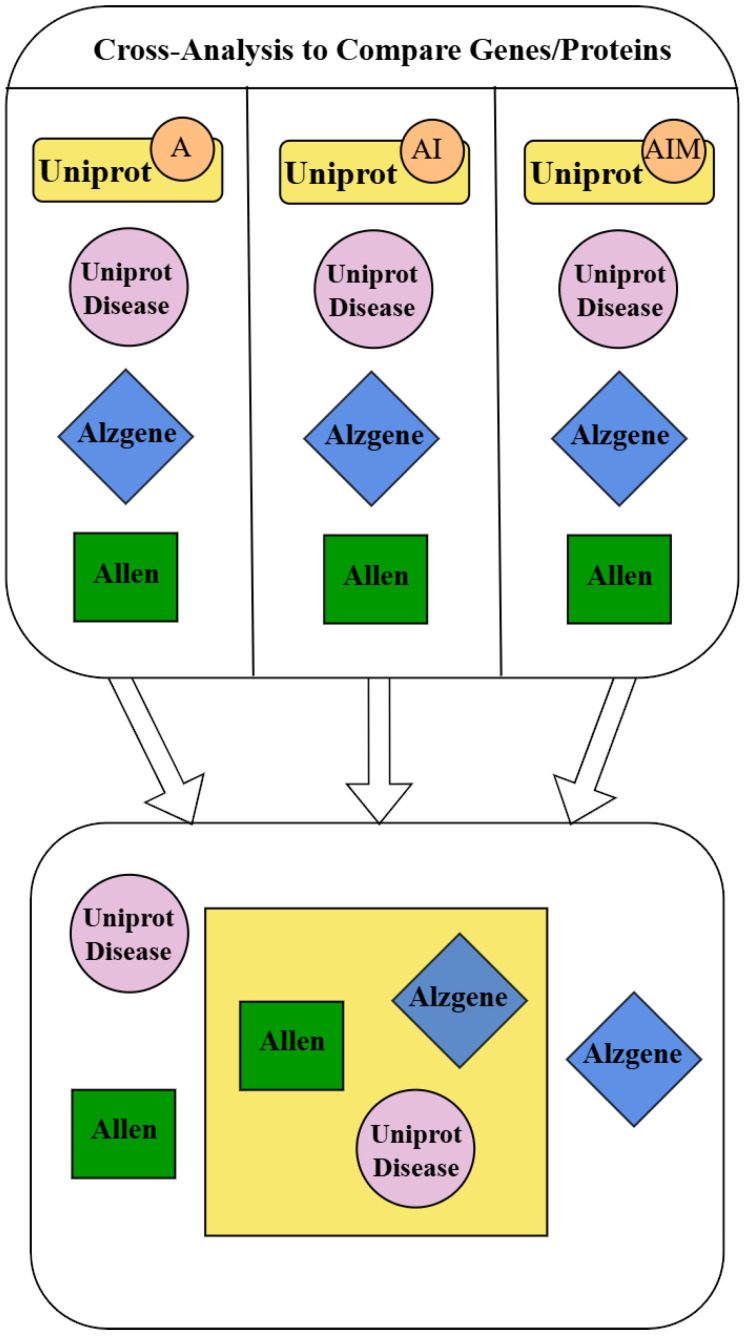
Workflow of the procedure of the dataset search and matching. Each dataset obtained by the keyword search of UniProt was matched with protein lists retrieved from the other databases. The matching yielded new datasets that collected proteins present in both UniProt and other databases (symbols in the yellow square), and non-overlapping protein datasets (symbols outside the yellow square). UniProt keywords: A (Alzheimer), AI (Alzheimer AND iron), AIM (Alzheimer AND iron AND mitochondrial).

The cross-analysis of the data downloaded from the different databases produced lists of overlapping proteins and distinctive proteins. More precisely, each of the three datasets obtained from keyword searches in UniProt (UniProt A, AI, and AIM) were compared with datasets obtained from UniProt Disease, AlzGene, and AHBA databases. Overlapping proteins were found after matching by Microsoft Access software, and these proteins are the most interesting result of our data mining procedure because they have been identified in different types of databases, which relied on heterogeneous evidence such as gene expression, gene ontology, and function. Proteins retrieved from multiple databases can be strongly considered as proteins involved in iron and mitochondrial impairment associated with AD. Other proteins were described as AD-related only in a single database. The procedure followed in our study is summarized in Figure 1.

To identify the proteins that stand out as the most interesting result of our research, we classified them according to the number of datasets in which they were present. We checked whether the proteins of the three UniProt categories (UniProt AIM, UniProt AI, and UniProt A) were also present in the other datasets. The number of overlaps was calculated and generated a ranking of biological interest. This classification is shown in Table 1.

Furthermore, all the proteins in the UniProt AIM dataset are particularly relevant regardless of their overlap, as they represent the focus of our investigation, i.e., the goal of identifying proteins modulated by iron and mitochondrial metabolism in AD. Interestingly, three proteins of this group were spotted by our biochemical analysis of the dataset obtained after the search of UniProt with the keywords “heme synthesis AND mitochondrial”; as mentioned above, these proteins were PICALM, appoptosin, and humanin. In addition, three other proteins of this most important dataset UniProt AIM are as follows:

- Amyloid-beta precursor protein (APP, gene: APP) is a precursor that, upon proteolytic processing, generates the amyloid-beta (Aβ) peptide, which is the main constituent of amyloid plaques implicated in the etiology of AD [31];
- Alpha-synuclein (gene: SNCA) is a protein involved in the regulation of pre-synaptic function and the release of neurotransmitters, which in the phosphorylated form has a higher propensity for aggregation and formation of neurotoxic fibrils not only in Parkinson’s disease but also in AD [32];
- Prostaglandin G/H synthase 1 (COX-1, gene: PTGS1) is an enzyme that transforms arachidonate to pro-inflammatory prostanoids; its expression is increased in AD, and the nonsteroidal anti-inflammatory drugs (NSAIDs) that inhibit COX slow the progression and delay the onset of AD [33].

Finally, many proteins remained excluded from overlapping because they were extracted from a single database. Among these, we highlight the aforementioned proteins of biochemical interest found in the analysis of heme synthesis: mitoferrin 1 and 2, and frataxin.

**Figure 2:**
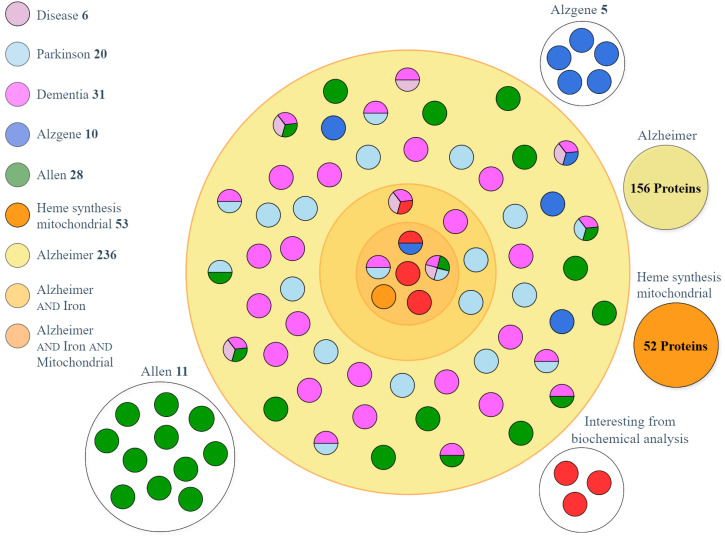
Graphical representation of the results of dataset matching. Except for AlzGene and AHBA, all datasets were obtained from UniProt, and the corresponding color codes are shown in the legend, with the number of listed proteins. The overlapping proteins (single-color and multicolored circles) are distributed across the three UniProt datasets: A (outer circle), AI (middle circle), and AIM (inner circle). Multicolor circles represent proteins retrieved from multiple datasets.

A graphical representation of the results of the matching between datasets is shown in Figure 2, which builds an overview of the proteins present in the three UniProt datasets AIM, AI, and A, and shows the presence of the proteins extracted only from individual databases. Figure 2 highlights the overlaps between databases and the uniqueness of each dataset; in fact, the proteins shared by multiple datasets are represented as single-color or multicolor circles spread across a circular area, which contains the proteins selected from UniProt and matching with other datasets. Outsider proteins have been identified only from single databases. Figure 2 outlines the distribution of the most interesting proteins; the multicolor circles represent the proteins listed in Table 1, and the inner circular area contains the most promising proteins identified in this study, which is focused on the retrieval of potential markers of iron and mitochondrial defective AD. All proteins are gene-name-tagged in Supplementary Figure S1.

The most interesting proteins revealed by our data mining and matching approach were retrieved either with the UniProt AIM search, or by multiple dataset matching, or they have been identified by the biochemical analysis of “heme synthesis AND mitochondrial” search results. These proteins are promising markers of AD related to iron and mitochondrial dysmetabolism, and are summarized in Table 2. Also, the search by GO terms confirmed the involvement of several proteins of this list in iron or mitochondrial function or AD pathology. The investigation of amino acid composition revealed that most of these proteins had a high E/Q ratio; if this value is used as an index of sensitivity to tissue oxygenation, we can conclude that we selected a dataset of proteins highly dependent on well-oxygenated neurons for their synthesis.

## 3. Discussion

This work proposes a new method of data search that combines information from the datasets of genes and proteins with the aim of finding diagnostic markers.

Information about protein features linked to disease onsets is scattered across various data sources and is not sufficiently integrated to be effectively utilized in a linear data mining approach to biomarker discovery. Several strategies have been proposed to integrate information, depending on the aim of each research. The most common approach to biomarker identification consists of data mining from a single database (UniProt is used as the main proteomic resource), followed by bioinformatic analysis [47]. Another strategy is the text mining procedure [48]. Our study proposes the combined data mining of several databases that overcomes many of the limitations of querying a single database, and it is similar in its principle to what previously proposed by other researchers [49]; however, our approach improves the selection of databases by combining UniProt with AHBA, which contains information about genes and codified proteins obtained in well-defined areas of the human brain. Importantly, we did not use a standard pathway knowledge tool to define the relevance of our results, but we carried out a biochemical analysis of each protein extracted by database matching; we believe that a specific biochemical identification of protein functions performs better in a stringent query like the search for biomarkers in AD related to iron and mitochondrial impairment. As a proof-of-concept (POC) study, we sought to achieve the identification of novel markers for AD. We focused on the pathology caused by mitochondrial damage associated with, or resulting from, iron accumulation. While few protein markers of AD (biomarkers of amyloid-beta (Aβ) deposition and of tau-protein-related hyperphosphorylation) are recognized and used in both basic and clinical research in neurodegenerative diseases [50], research needs new biomarkers to better understand the onset and progression of the disease, as well as the complex cascade of biochemical mechanisms that are triggered depending on the stage and the interactions between different pathological processes. It has been reported that the mitochondrial dysmetabolism and the perturbation of iron homeostasis are contributing causes of disease [38,51], and have a negative prognostic value in the progression of AD [52,53]; therefore, in this study, we created a procedure to obtain a new panel of proteins that can be validated in the future as specific markers of AD, potentially exploitable both in the prognosis and follow up of the disease.

With this focus, several databases were searched by means of specific keywords and dataset matching, yielding a list of proteins of particular interest.

Some proteins have been found to be present in multiple databases and are particularly relevant because they are the result of cross-referencing between very different databases, based on gene expression data, protein function data, and the meta-analysis of clinical data. Indeed, some of these proteins are well-recognized markers of this disease, and were convincingly selected by our method (APP [31], alpha-synuclein/SNCA gene [32], tau/MAPT gene [34], ApoE [54]); in addition, many other proteins of this list warrant further investigation and validation as biomarkers. These proteins, shown in Table 1, were also investigated by a PubMed search “protein name AND Alzheimer”. This evaluation underlined the role of these proteins in AD onset and supported their potential value as biomarkers. In addition to the proteins described in the Section 2 (PICALM, gene: PICALM; APP, gene: APP; alpha-synuclein, gene: SNCA), the involvement of the proteins listed in Table 1 in AD pathology can be highlighted as follows:

- ApoE (gene: APOE): the APOE4 gene is the strongest genetic risk factor for the development of LOAD [54].
- Presenilins (gene: PSEN): presenilins form the catalytic core of γ-secretase complexes that cleave a transmembrane fragment of APP to release Aβ [35,36].
- ATP-binding cassette transporter A7 (gene: ABCA7): a susceptibility factor of LOAD. The reduction in ABCA7 expression or loss of function increases amyloid production [37].
- Disintegrin and metalloproteinase domain-containing protein 10 (gene: ADAM10): transmembrane metalloproteinase responsible for alpha-secretase cleavage of APP. The non-amyloidogenic APP processing by ADAM10 is decreased in AD [39].
- Drebrin (gene: DBN1): is involved in memory-related synaptic plasticity in the hippocampus. AD brains show remarkable reductions in drebrin immunoreactivity [40].
- Ubiquitin carboxyl-terminal hydrolase isozyme L1 (Gene: UCHL1): regulates APP processing by promoting BACE1 degradation, its downregulation increases APP [41].
- NADH-ubiquinone oxidoreductase chain 1 (gene: MT-ND1): core subunit of the mitochondrial membrane respiratory chain Complex I. Mitochondrial gene transcript is altered in AD [42].
- Sortilin-related receptor (gene: SORL1): because it is a sorting receptor for APP, regulating its intracellular trafficking and processing into amyloidogenic-beta peptides, SorL1 deficiency is a genetic predisposition to AD. SorL1 depletion leads to the disturbance of iron homeostasis in the rat hippocampus, mitochondrial oxidative stress, hippocampal degeneration, and impaired spatial memory [43].
- Kallikrein-6 (gene: KLK6): serine protease that degrades alpha-synuclein and prevents its polymerization. The dysregulation of kallikreins has been linked to several neurological disorders, including AD [44].
- RE1-silencing transcription factor (gene: REST): a transcriptional repressor of neuronal genes in neural stem cells. The deletion of REST in mouse models accelerates neurodegeneration and cognitive decline by increasing Aβ deposition and the accumulation of misfolded and phosphorylated tau [45].
- Sequestosome-1 (gene: SQSTM1): a multifunctional scaffolding protein that plays a central role in autophagy. Genetic association studies have reported that it may play an important role in the progression of AD via associations with Aβ levels in cerebrospinal fluid and Aβ deposition in the brain of patients with AD [46].

In addition to the results of dataset matching, we paid particular attention to the UniProt AIM dataset, which is the collection most focused on the correlation between iron, energy dysmetabolism and AD. Six proteins are particularly interesting (proteins that are represented in the inner circle of Figure 2): PICALM, appoptosin, humanin, APP, alpha-synuclein, and prostaglandin G/H synthase 1 (COX-1).

Finally, among the proteins involved in heme iron homeostasis, we have highlighted the following: mitoferrins and frataxin.

Altogether, by this novel approach, we were able to create a data set of possible markers of AD proteins, and we summarized this list in Table 2. Most of these proteins have been investigated in human or animal studies or in in vitro models of AD, but notwithstanding their established involvement in the pathology, their value as biomarkers still awaits future validation. Because this study was focused on the identification of protein markers and was not refined by data of differential gene expression, we could not directly corroborate the theoretical results; however, we attempted to check whether the selected proteins could be modulated in hypoxic brain tissue, and we analyzed the amino acid composition in terms of the E/Q ratio. Theoretically, the proteins with a high value of E/Q could be good markers of AD due to environmental conditions hampering their synthesis and activity. In the list of proteins selected by our data mining and matching approach (Table 2), the majority have a high E/Q ratio, greater than 1, and in many cases, are extremely high, up to a value of 3.4. Considering that in our previous analysis of epidermal proteins, we reported an E/Q median value of 0.5 for hypoxic proteins and a median value of E/Q ratio greater than 1 for oxygenated proteins [21], we could hypothesize that several proteins identified in our study could be poorly synthesized in hypoxic, mitochondrial defective, and iron overloaded, brain tissue. Indeed, we have evidence that some of these proteins are downregulated in AD [39,40,55,56,57,58]. In this study, this preliminary analysis simply substantiates the outcome of our data mining and matching approach. In future investigation, although the ratio E/Q is not always directly proportional to protein expression because other molecular mechanisms can prevail, potential AD markers could be validated combining the E/Q information with the knowledge about iron accumulation [59,60] and with anatomical details of hypoxic brain regions.

Further studies of differential expression must confirm the proteins identified in this study as new markers of AD, especially possible markers of the pathology driven by energy deficit and iron dysmetabolism. Future experimental studies or the investigation of gene expression databases can compare the expression of potential biomarkers, alone or in combination, in the healthy and AD population, and can establish the optimal utilization of these biomarkers. In addition, the data collection performed in this study can be used to identify the brain areas where these proteins are most expressed, which consequently would be regions most critically involved in the development of AD. To this end, the use of the AHBA database could be particularly useful, because it allows for the spatial localization of the genes (and encoded proteins) identified by our research. These brain areas could be the subject of further analysis.

### Limitations

There are some limitations in this work that should be considered.

First, our data mining and matching were obtained mainly by computational methods, without confirmation in cellular, animal, or human models, so there is a need for experimental validation with future works or supplementary analyses.

Second, this work aimed to find the most promising proteins that could be biomarkers in AD via cross-reference and biochemical qualitative profiling, but it lacks descriptive statistics or statistical tests that can lend quantitative robustness to the findings, limiting the strength of the inferences.

Third, comparing different databases is interesting for searching new biomarkers, but it runs the risk of introducing bias into the analysis due to the heterogeneity of the data. In our work, UniProt is a highly reliable database; the AHBA and AlzGene could have some limitations. UniProt is a database that is annotated, verified, and manually curated by experts. In addition, it acts as a central hub, connecting to other resources through regularly updated cross-references to databases like PDB, DDBJ/ENA/GenBank, Ensembl, and RefSeq. The AHBA is limited by a relatively small sample size, so it can introduce some limitations in terms of generalizability. However, it is the result of an external advisory council of experts in human neuroanatomy, imaging, genetics, genomics, and ontologies [61]. AlzGene is part of the Alzheimer’s Research Forum Web site (www.alzforum.org, accessed on 2 May 2025). It catalogs all genetic association studies in the field of Alzheimer’s disease, conducting a systematic meta-analysis of polymorphisms with genotype data available in three or more independent case–control samples. It has been updated until 2011; therefore, it does not contain the most recent studies on the topic. However, the selection of genes was carried out considering a very strict threshold (*p* < 5.0 × 10^−8^).

Finally, the chosen method of investigation does not involve gene expression analysis, nor a comprehensive meta-analysis of the literature. The results are limited to theoretical findings, which should be confirmed in future studies. For example, the E/Q ratio could be of interest by analogy with the results of the study previously published, which have demonstrated the relationship in the skin and liver [21]. However, no clear evidence could be reported in this data mining process. E/Q analysis could be exploited in future studies in combination with the knowledge about iron accumulation and anatomical information. Particularly, some regions with a high probability of iron accumulation (putamen, globus pallidus, caudate nucleus, thalamus) could be investigated in terms of E/Q.

## 4. Materials and Methods

### 4.1. Data Sources

We used data from two research databases: the database UniProt (https://www.uniprot.org/, accessed on 2 May 2025) and the AHBA (https://portal.brain-map.org/, accessed on 2 May 2025); we added the results of a query in the literature database AlzGene (https://www.alzforum.org/alzgene, accessed on 2 May 2025). Then, we cross-referenced these results with a search in the free search engine on life sciences and biomedical topics PubMed (https://pubmed.ncbi.nlm.nih.gov/, accessed on 2 May 2025).

In our search in the protein database UniProt, we used protein data from the UniProtKB/Swiss-Prot subsection (Knowledgebase, https://www.uniprot.org/uniprotkb, accessed on 2 May 2025), which contains verified and manually annotated entries by experts (Reviewed). In addition, it acts as a central hub by connecting to other resources through regularly updated cross-references to databases like PDB, DDBJ/ENA/GenBank, Ensembl, and RefSeq. The data downloaded were reviewed, human, and searched in the following fields: Entry, Entry name, Protein names, Gene names (Primary), Function, Length, Sequence, Gene Ontology IDs, Gene Ontology (biological process), Gene Ontology (cellular component), Gene Ontology (molecular function), and Gene Ontology (GO).

The AHBA [23] provides online open-source resources about the genes, gene expression, and neuroanatomical information of different groups of subjects. We downloaded “Technical white paper: complete list of genes characterized by in situ hybridization in adult human brain studies” from https://community.brain-map.org/t/documentation-human-brain-atlas/2879, document “Gene List” (https://community.brain-map.org/uploads/short-url/bMXzw0nEZUer7Dw2T8Sb1mxP3fN.pdf, both accessed on 2 May 2025), October 2012 v.2, and referred to the section Table 1. The genes were characterized by ISH in the 1000 gene survey in the cortex (Cortex Study). We used data from the Cortex table, as it is more general. We used the column Disease, using the keyword “Alzheimer” to select all AD genes.

The AlzGene site (https://www.alzforum.org/alzgene, data downloaded on June 2023) contained the section “Top Result”, developed to facilitate the identification of genes with the strongest genetic associations with AD, based on available meta-analyses. A copy of “Top Results” is archived at the page https://web.archive.org/web/20230603235811/http://www.alzgene.org/TopResults.asp (accessed on 29 May 2025).

From both the AHBA and AlzGene databases, we downloaded gene datasets, and their codified proteins were retrieved from UniProt after a search for Gene Name. We selected the proteins of interest in the “Gene Names (Primary)” field of UniProt output.

The PubMed website was used to search for specific papers based on results emerging from the UniProt, AHBA, and AlzGene searches.

**Figure 3:**
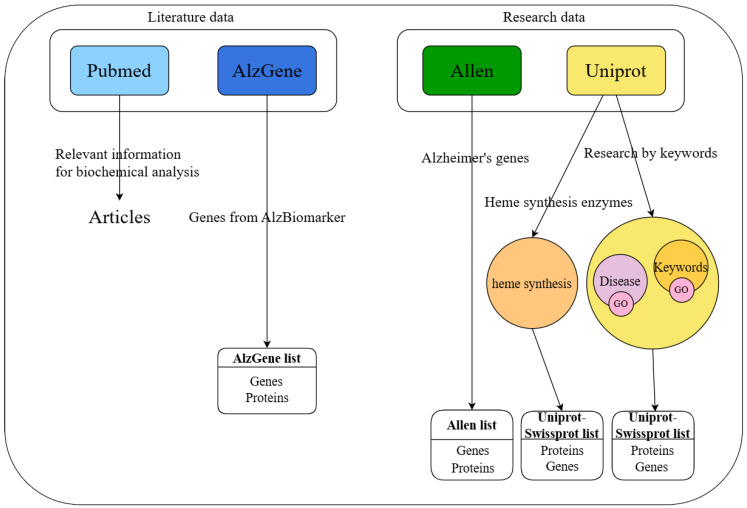
Data collection from the research and literature databases. Color depicted as in Figure 2 caption.

The workflow of data collection is depicted in Figure 3.

### 4.2. Database Investigation

#### 4.2.1. UniProt Investigation from Disease Section

Among the diseases listed in UniProt Disease, AD has a dedicated section that compiles data on proteins implicated in its pathogenesis (https://diseases.uniprot.org/disease/DI-03832/protein, accessed on 2 May 2025). Specifically, UniProt Disease categorizes AD-related proteins based on their association with EOAD and LOAD forms of the disease. We downloaded three proteins associated with EOAD and three proteins associated with LOAD (Supplementary Table S1).

#### 4.2.2. UniProt Investigation with Keywords

We downloaded the AD-related genes from the UniProt site using appropriate keywords to produce lists of human-reviewed proteins. In the UniProt homepage, for the section “Find your protein”, we chose UniProtKB from the drop-down menu and typed the search keyword. Then, we selected “Reviewed” as Status and “Human” as Popular Organisms on the page of the UniProtKB results. All the results were downloaded by selecting “Download all”, Format “Excel”, Compressed “No”, and customizing columns by selecting the fields Entry name, Protein names, Gene Names, Organism, Gene names (Primary), Length, Sequence, Reviewed, and Involvement in disease. The Entry field was added automatically to the table generated. We used a combination of the following keywords: with the “Alzheimer” keyword alone, we extracted a list of 236 proteins (Supplementary Table S2); with the “Alzheimer” and “iron” keywords combination, we extracted a list of 25 proteins (Supplementary Table S3); finally, with the keywords “Alzheimer” and “iron” and “mitochondrial”, we extracted a list of 16 proteins (Supplementary Table S4).

Furthermore, we customized the search with keywords by adding the following columns: Gene Ontology IDs, Gene Ontology (biological process), Gene Ontology (cellular component), Gene Ontology (molecular function), and Gene Ontology (GO). In the GO terms, we then searched for single keywords “Alzheimer”, “iron,” and “mitochondrial” (Supplementary Table S5). We identified 4 proteins by “Alzheimer” GO term, 8 proteins by “iron” GO term, and 34 proteins by “mitochondrial” GO terms, and we verified that they were present in the output of UniProt search by keywords. This final analysis checked that our data mining strategy did not miss any protein related to AD.

#### 4.2.3. UniProt Investigation by Keywords Relative to Similar Cognitive Pathologies

We extracted a list of 20 proteins using the keywords “Parkinson AND Alzheimer” in UniProt (Supplementary Table S6). Moreover, we obtained a list of 31 proteins using the keywords “Dementia AND Alzheimer” in UniProt (Supplementary Table S7).

#### 4.2.4. UniProt Investigation of the Heme Synthesis Pathway

We looked for proteins related to heme synthesis and mitochondrial function by searching with the keyword “heme synthesis mitochondrial” and selecting “Human”, “Reviewed”. We downloaded a list of 53 proteins (Supplementary Table S8). Furthermore, to investigate the whole synthesis pathway, we searched the information available in UniProt about each biosynthetic enzyme. The eight enzymes of this biosynthetic pathway were downloaded from the Kegg Pathway database (https://www.kegg.jp/module/M00868, accessed on 2 May 2025), and the relative information was downloaded from UniProt; the AA relative frequencies were calculated for each enzyme involved in heme synthesis (Supplementary Table S9). We also calculated the E/Q ratio to assess which proteins were the most sensitive to oxygen.

#### 4.2.5. PubMed Investigation

The two protein lists described in Section 4.2.2 and Section 4.2.4 were further investigated in PubMed to highlight their role in AD using an extensive search of the database and an exhaustive review of the extracted papers (reported in the Section 3 and References section).

#### 4.2.6. AlzGene Investigation

The AlzGene database was searched for AD biomarkers, and a list of ten proteins was downloaded from the “Top Result” section (Supplementary Table S10, downloaded on June 2023). Then, it was filtered by significance (<0.00001) of evidence (for genome-wide significant findings, *p* < 5.0 × 10^−8^). All results were assessed and ranked for their epidemiological credibility using two methods: the HuGENet interim criteria (taking into account the amount of evidence and consistency of replication) and Bayesian analyses (a log_10_ Bayes Factor ≥ 5 suggests that the data increase over 100,000-fold the odds of a given finding being true vs. not true, is considered as strong support in the context of genetic association studies). To map the gene with the relative protein, we used UniProt and the field Gene Names (primary).

#### 4.2.7. AHBA Investigation

We identified 28 proteins of genes related to AD from the AHBA database (Supplementary Table S11). To map the gene with the relative protein, we used UniProt and the field Gene Names (primary).

### 4.3. Analysis of Amino Acid Content

The AA content of the selected proteins was downloaded by ProtParam, a tool that allows for the computation of various physical and chemical parameters for a given protein stored in UniProtKB (https://web.expasy.org/protparam/, accessed on 2 May 2025). To analyze the composition of proteins, we calculated the amino acid (AA) content in terms of relative frequency and the ratio E/Q (Glu/Gln) using a tool “GPLAB-Gene and Protein Virtual Lab” (https://gplab.diff.org, Version 1.1.25, accessed on 2 May 2025), which allows for the computation of AA content and their ratio, as previously published [62]. The ratio E/Q can be considered an indicator of sensitivity to oxygen [21].

### 4.4. Data Matching

As a final analysis, we investigated overlapping and singularities among tables downloaded in previous steps (Tables S1–S11). Each table was imported, analyzed, and connected using the relational database software Microsoft Access for Microsoft 365 MSO (Version 2505 64-bit). To identify overlaps between tables, we used the queries with the INNER JOIN operator, while singularities were detected using LEFT or RIGHT JOIN, in combination with the IS NULL condition. The relationship between the tables was established through the field Gene Names (primary). All subset results were collected and are presented in a summary table to provide an organic overview of the data (Supplementary Table S12).

## 5. Conclusions

This work proposes a novel approach to search and harmonize information from the datasets of genes and proteins, with the aim of finding potential new clinical biomarkers. As a POC study, we searched for markers for AD, but the workflow can be extended to many other diseases.

With this new integrated method of data mining, data matching, and biochemical analysis, we obtained a dataset of proteins that are altered in AD when the cognitive deficit is associated with the neuronal energy deficit exacerbated by iron accumulation. This work can be preliminary to expression studies, which will validate and suggest optimal combinations of these potential biomarkers.

## Figures and Tables

**Table 1:** Overlapping proteins. The datasets UniProt AIM, AI, and A were matched with the other datasets, and several proteins were found in multiple datasets.

Overlapping Identification	AIM ^1^	AI ^2^	A ^3^
5 datasets	APP	0	0
4 datasets	0	APOE	MAPT
			PSEN1
			PSEN2
			ABCA7
3 datasets	PICALM	0	ADAM10
	SNCA		DBN1
			UCHL1
			MT-ND1
			SORL1
			KLK6
			REST
			SQSTM1

UniProt was searched with the following keywords: ^1^ Alzheimer, iron, and mitochondrial (AIM); ^2^ Alzheimer and iron (AI); ^3^ Alzheimer (A).

**Table 2:** Features of the protein dataset resulting from the cross-analysis. The glutamate (E)-to-glutamine (Q) ratio is considered an index of oxygen dependence.

Gene Name (Primary)	Entry Name ^1^	Protein Name	E/Q	Dataset ^2^	GO ^3^ Term	Role in AD
**PICALM**	**PICAL_HUMAN**	**PICALM**	0.862	3	I	[24]
**SLC25A38**	**S2538_HUMAN**	**Appoptosin**	0.571	2	M	[25]
**APP**	**A4_HUMAN**	**APP**	2.556	5	A	[31]
**SNCA**	**SYUA_HUMAN**	**Alpha-synuclein**	3.000	3	I,M	[32]
**PTGS1**	**PGH1_HUMAN**	**COX-1**	1.333	2	-	[33]
MAPT	TAU_HUMAN	MAPT	1.788	4	M	[34]
PSEN1	PSN1_HUMAN	Presenilin-1	1.684	4	M	[35]
PSEN2	PSN2_HUMAN	Presenilin-2	3.000	4	M	[36]
ABCA7	ABCA7_HUMAN	ABCA7	1.384	3	-	[37]
APOE	APOE_HUMAN	Apolipoprotein E	1.250	4	-	[38]
ADAM10	ADA10_HUMAN	ADAM10	1.294	3	-	[39]
DBN1	DREB_HUMAN	Drebrin	2.676	3	-	[40]
UCHL1	UCHL1_HUMAN	UCHL1	1.833	3	-	[41]
MT-ND1	NU1M_HUMAN	MT-ND1	1.833	3	M	[42]
SORL1	SORL_HUMAN	SorL1	1.595	3	-	[43]
KLK6	KLK6_HUMAN	Kallikrein-6	0.786	3	-	[44]
REST	REST_HUMAN	REST	2.125	3	-	[45]
SQSTM1	SQSTM_HUMAN	Sequestosome-1	3.417	3	M	[46]
SLC25A37	MFRN1_HUMAN	Mitoferrin-1	0.765	1	-	[27]
SLC25A28	MFRN2_HUMAN	Mitoferrin-2	1.063	1	-	[28]
FXN	FRDA_HUMAN	Frataxin	1.429	1	-	[29]

^1^ Protein unique identifier from UniProt. ^2^ Number of datasets containing the protein. ^3^ Proteins retrieved by GO term keywords iron (I), mitochondrial (M), and Alzheimer (A). Proteins in bold were retrieved by UniProt keyword search Alzheimer, iron, and mitochondrial (AIM).

## Data Availability

The original contributions presented in this study are included in the article/Supplementary Material. Further inquiries can be directed to the corresponding author(s).

## Supplementary Materials

The following supporting information can be downloaded at: https://www.mdpi.com/article/10.3390/ijms26157536/s1.

## Abbreviations

The following abbreviations are used in this manuscript:

AD	Alzheimer’s disease
E/Q	Glutamate/glutamine
GO	Gene Ontology
