# Supplementary figures and images for: Data Mining and Biochemical Profiling Reveal Novel Biomarker Candidates in Alzheimer’s Disease
Source: Int J Mol Sci. 2025 Aug 4;26(15):7536. doi: 10.3390/ijms26157536 (PMC12347173; doi:10.3390/ijms26157536)

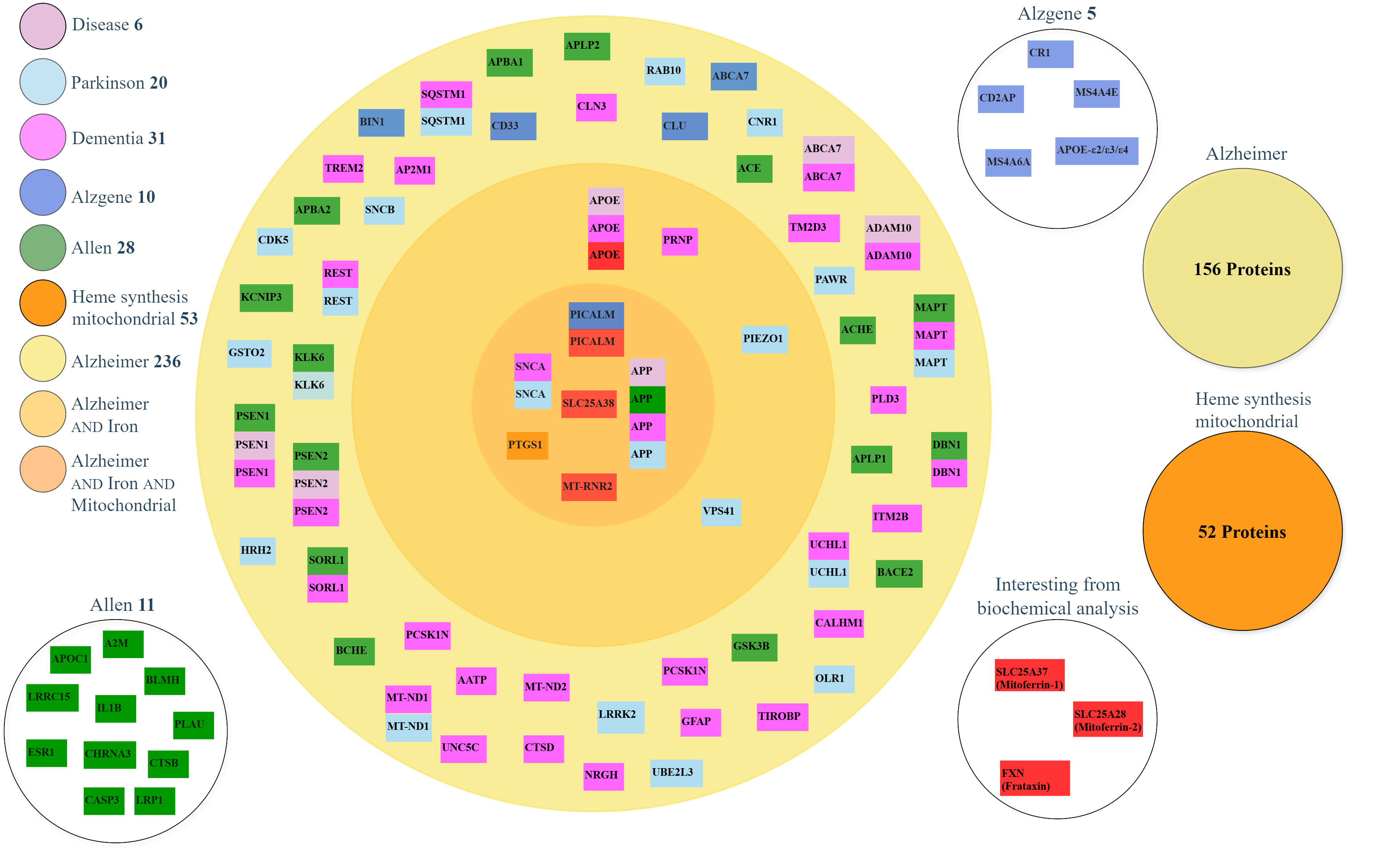

Supplement: Supplementary file 1 [file ijms-26-07536-s001.zip › Figure S1.jpg]
